# Ecology of asynchronous asexual replication: the intraerythrocytic development cycle of *Plasmodium berghei* is resistant to host rhythms

**DOI:** 10.1186/s12936-021-03643-z

**Published:** 2021-02-19

**Authors:** Aidan J. O’Donnell, Sarah E. Reece

**Affiliations:** Institute of Evolutionary Biology, and Institute of Immunology and Infection Research, School of Biological Sciences, University of Edinburgh, Charlotte Auerbach Rd, Edinburgh, EH9 3FL UK

**Keywords:** Periodicity, Synchrony, Circadian rhythm, Feeding timing, Intraerythrocytic development cycle, Asexual replication, Gametocyte, Transmission, Fitness

## Abstract

**Background:**

Daily periodicity in the diverse activities of parasites occurs across a broad taxonomic range. The rhythms exhibited by parasites are thought to be adaptations that allow parasites to cope with, or exploit, the consequences of host activities that follow daily rhythms. Malaria parasites (*Plasmodium*) are well-known for their synchronized cycles of replication within host red blood cells. Whilst most species of *Plasmodium* appear sensitive to the timing of the daily rhythms of hosts, and even vectors, some species present no detectable rhythms in blood-stage replication. Why the intraerythrocytic development cycle (IDC) of, for example *Plasmodium chabaudi,* is governed by host rhythms, yet seems completely independent of host rhythms in *Plasmodium berghei,* another rodent malaria species, is mysterious.

**Methods:**

This study reports a series of five experiments probing the relationships between the asynchronous IDC schedule of *P. berghei* and the rhythms of hosts and vectors by manipulating host time-of-day, photoperiod and feeding rhythms.

**Results:**

The results reveal that: (i) a lack coordination between host and parasite rhythms does not impose appreciable fitness costs on *P. berghei*; (ii) the IDC schedule of *P. berghei* is impervious to host rhythms, including altered photoperiod and host-feeding-related rhythms; (iii) there is weak evidence for daily rhythms in the density and activities of transmission stages; but (iv), these rhythms have little consequence for successful transmission to mosquitoes.

**Conclusions:**

Overall, host rhythms do not affect the performance of *P. berghei* and its asynchronous IDC is resistant to the scheduling forces that underpin synchronous replication in closely related parasites. This suggests that natural variation in the IDC schedule across species represents different parasite strategies that maximize fitness. Thus, subtle differences in the ecological interactions between parasites and their hosts/vectors may select for the evolution of very different IDC schedules.

## Background

Biological rhythms are a ubiquitous feature of life that enable organisms to coordinate with environmental rhythms, such as those caused by the Earth’s rotation (‘circadian’ rhythms). Parasites from diverse taxa couple their activities to daily rhythms in the within-host environment, the activity patterns of hosts and vectors, as well rhythms in the abiotic environment [1, 2]. Some rhythmic activities are thought to enhance transmission, such as rhythms in the migration of filarial worm larvae (*Wuchereria bancrofti)* from the host’s deep tissues to the peripheral blood capillaries at the time-of-day its vector forages for blood [3], and the coccidian parasite *Isospora turdi,* sheds at the time-of-day that minimizes mortality from UV exposure [4]. Other rhythmic activities are thought to enable parasites to cope with challenges imposed by, and exploit opportunities provided by, rhythms in the within-host environment. For example, *Botrytis cinerea,* a fungal pathogen of *Arabidopsis thaliana*, has canonical clock (transcription–translation-feedback-loop) controlled rhythms in virulence that enable it to overwhelm hosts defenses when they are upregulated in the evening [5, 6]. Similarly, *Trypanosoma brucei* uses a clock of unknown components to control gene expression of over two hundred of its genes, potentially allowing it to coordinate its metabolism with that of the host [7].

Malaria parasites are renowned for their rhythmicity, for example, the species of parasite infecting a patient can be diagnosed from the regularity of fevers [8]. Fevers are caused by the synchronous bursting (schizogony) of asexually replicating blood stage parasites when they complete an intraerythrocytic development cycle (IDC). Human malaria parasites have IDC durations of 24, 48, and 72 h and so, cause fever with matching periodicity. The rodent malaria parasite species *Plasmodium chabaudi* also exhibits a synchronous IDC (lasting 24 h) and schizogony is timed to coincide with processes related to the host’s daily feeding pattern [9–11]. Specifically, IDC completion coincides with the appearance of the amino acid isoleucine in the blood (as a product of the host digesting its food), which is a nutrient the parasite must acquire from the host’s food [12]. Scheduling the IDC around the availability of necessary resources is consistent with the observation that experimentally mismatching the IDC with host circadian rhythms (which dictates host activity and foraging rhythms) results in the disruption of important cellular processes, and a loss of both asexual stages and gametocytes [1, 13–15]. Furthermore, the IDC schedule determines gametocyte age at the time-of-day the mosquito vector forages for blood [16] and gametocytes exhibit time-of-day variation in their infectiousness to mosquitoes [17]. For *P. chabaudi,* it appears that by completing the IDC at night—the time-of-day that nocturnal rodents forage—asexual replication is most successful and gametocytes are also at their most infective age when vectors blood feed [17, 18].

**Fig. 1:**
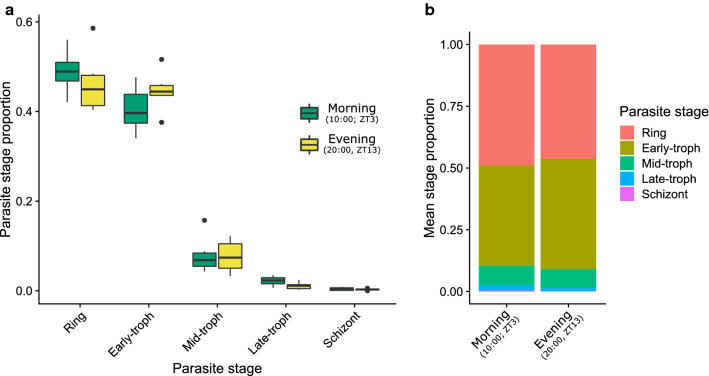
*Plasmodium berghei* has an asynchronous IDC. Figure created from data in O’Donnell 2019 [49]. The proportion of parasites at each IDC stage does not differ significantly between morning (10:00 UTC; ZT3) and evening (20:00 UTC; ZT13), $\chi_{4}^{2}$ = 5.83, P = 0.21. Shown in (**a**) are median parasite stage proportions (black line) and 25–75 percentiles, with whiskers 1.5 times the interquartile range and dots representing outliers. Shown in (**b**) is the mean parasite stage proportion. Note, rings, early trophozoites (troph) and mid trophozoites compose the majority of stages observed in peripheral blood, with late trophozoites and schizonts present but in reduced numbers due to sequestration to deep tissues [3, 49, 50]. Infections (n = 6) were sampled on Day 5 PI from wild type MF1 mice in a standard 12 h light: 12 h dark photoperiod

Despite the benefits of an IDC in which parasites develop synchronously and transition between developmental stages at particular times of day, not all malaria parasite species are synchronous. Specifically, two of the four rodent malaria parasites, *Plasmodium berghei* and *Plasmodium yoelii*, have IDCs that are not a multiple of 24 h (21–23 h [19–21] and 18 h, respectively [22]), and are developmentally asynchronous, with all IDC stages occurring simultaneously in the blood throughout the host’s circadian cycle (Fig. 1). Whilst the IDC schedules of *P. berghei* and *P. yoelii* are well-studied, observations suggest an asynchronous IDC occurs in some parasite species of birds [23] and lizards [24], suggesting it is a taxonomically diverse trait. Whether an infection exhibits synchronous or asynchronous replication is not dictated purely by the host because, for example, the opposing IDC schedules of *P. berghei* and *P. chabaudi* are apparent when each species infects the same age, sex, and strain of laboratory mice. This observation, coupled with recent discoveries of parasite control of the IDC schedule [25–27] suggests the degree of synchrony and the timing of the IDC are at least in part controlled by parasites. If so, it raises the possibility that a synchronous or asynchronous IDC are different parasite strategies that have evolved by natural selection because they enhance parasite fitness [18]. Synchronous or asynchronous replication is unlikely to have evolved as a consequence of abiotic environmental differences because *P. berghei* and *P. yoelii* are found in different climates (*P. berghei* in the cool African highlands, *P. yoelii* in the warmer lowlands) [28]. Nor is it likely to be imposed by the mosquito vector as both the synchronous *Plasmodium vinckei* and the asynchronous *P. berghei* have been isolated from the same species of mosquito (*Anopheles dureni millecampsi*) [28]. Notably, *P. berghei* and *P. yoelii* prefer to infect reticulocytes whereas *P. chabaudi* is a generalist and *P. vinckei* is restricted to normocytes [28]. However, it is unlikely that red blood cell age preference imposes selection for a synchronous or asynchronous IDC for several reasons. First, the human malarias *P. vivax* and to a lesser extent *P. falciparum,* prefer reticulocytes but are predominantly synchronous [29, 30]. Second, reticulocytes are released into the blood in a circadian manner [31] so a synchronous IDC intuitively appears the best way to exploit reticulocytes. Third, Deharo et al. [21] do not find that invasion of normocytes or reticulocytes affects the IDC schedule of *P. berghei*. Explaining the evolution of a synchronous or asynchronous IDC requires knowledge of whether asynchronous species benefit from this style of IDC, and so asynchrony is an adaptation (i.e. confers fitness benefits), or whether asynchronous replication is costly but these species are governed by a constraint preventing them from synchronizing their IDC.

Here *P. berghei* (strain ANKA) is used as a model for asynchronous malaria parasites. It is well known that synchronized infections of *P. berghei* can be generated via laboratory methods (e.g. inoculations of pure merozoites [21]) but synchrony is rapidly lost within a few cycles [20]. Intriguingly there are reports of synchronous *P. berghei* experimental infections in ground squirrels (with schizogony occurring at the start of the active, feeding, period which is the inverse of the schedule of *P. chabaudi*) [18, 32] and in infections of laboratory mice in which hosts were subjected to “summer-like” lighting conditions (extended light hours with wide wavelength light) [33, 34]. Mammalian host physiological responses to summer photoperiods are thought to be controlled by the pineal gland [33], a regulator of host hormones such as melatonin, which has been implicated in modulating the rate of IDC development for synchronous species [35]. Based on these observations and knowledge of the ecological factors governing the IDC schedule of *P. chabaudi*, a series of five experiments were carried out to test whether IDC of *P. berghei* can be influenced by host rhythms and if asynchronous replication has fitness consequences. The experiments asked the following questions:

1. Does the time-of-day of blood stage infection affect the densities of asexual stages and gametocytes of *P. berghei*?
2. Does the IDC of *P. berghei* become synchronized in response to changes in photoperiod?
3. Do host feeding rhythms influence the IDC schedule of *P. berghei*?
4. Are there time-of-day differences in the circulating densities of gametocytes of *P. berghei*?
5. Are there time-of-day differences in *P. berghei* infectivity to mosquitoes?

## Methods

### Parasites, hosts, and vectors

Hosts were either wild type (WT) MF1 mice (experiments 1, 2, 4, 5) or *Per1/2*-null clock-disrupted mice previously backcrossed onto a C57BL/6J background for over 10 generations (experiment 3), all sourced from in-house breeding colonies. PERIOD 1 (PER1) and PERIOD 2 (PER2) are essential components of the core circadian clock (i.e. the transcription–translation-feedback-loop, TTFL) and *Per1/2*-null mice are arrhythmic when placed in constant darkness [36, 37]. All experimental mice were males, 8–10 weeks old, housed at 21 °C, and given unrestricted access to drinking water supplemented with 0.05% para-aminobenzoic acid (to supplement parasite growth, as is routine for this model system). Mice in experiments in which host light–dark and/or feeding schedules were altered (experiments 1, 2, 3) were entrained for at least 2 weeks prior to, and throughout the duration of infections. *Plasmodium berghei* (strain ANKA) was used to initiate all infections.

Parasites were administered via intraperitoneal injection (IP) at a dose of 1 × 10^6^ parasitized red blood cells (RBC) (experiments 1–4) or 1 × 10^5^ parasitized RBC (experiment 5). Infections were monitored by staining thin blood smears with 20% Giemsa for 20 min and asexual stages, gametocytes, IDC stage distributions (experiments 2 and 3) and gametocyte sex (experiment 4) were quantified via microscopy. Red blood cell densities were quantified using a Beckman Coulter Z2 particle counter. All infections were terminated on day 6 post infection (PI) to prevent host mortality due to complications from cerebral malaria. Parasites in all experiments were from same lineage recently transmitted through mosquitoes and cryopreserved within 1–2 passages of each other. Parasite stocks were then expanded in donor hosts before being used to initiate experimental infections. The IDC of *P. berghei* is asynchronous from frozen stocks and regardless of the number of passages between hosts.

*Anopheles stephensi* mosquitoes (experiment 5) were maintained under standard insectary conditions of 27 ± 1 °C, 70% humidity and 12 h light: 12 h dark photoperiod [lights on 07:00: lights off 19:00 UTC (Coordinated Universal Time)]. Female mosquitoes were randomly allocated to 2L holding cages (85 females per cage) and starved of fructose solution for 24 h before their blood meals. Each cage was exposed to an anaesthetized mouse for 30 min, all mosquitoes were able to blood feed until satiated and unfed females were removed from the cages (< 5 per cage in all cases). After feeding, mosquitoes were housed in incubators (humidity 60 ± 5%) at 20.5 ± 0.5 °C and 12 h light: 12 h dark photoperiod.

## Experimental designs and data collection

### Experiment 1: do host rhythms have fitness consequences for P. berghei?

This experiment tested whether the IDC of *P. berghei* aligns in a cryptic way with host rhythms in a manner beneficial to it, by comparing the performance of parasites in experimental infections stemming from donor hosts with either the same phase (timing) of host and parasite rhythms, or a 12 h difference. Experimental WT mice (n = 15 per treatment) were kept in a standard (lights on 07:00: lights off 19:00 UTC) or reverse photoperiod (lights off 07:00: lights on 19:00). On day 0 PI (08:00 UTC), parasites originating from donor mice housed in the standard photoperiod were used to simultaneously infect mice in both the standard and reverse photoperiods. This created a group of infections in which inoculated parasites entered hosts at the same time (phase) in their daily rhythms as the donor host they were collected from (‘matched’ parasites), and another group in which parasites entered hosts at the opposite phase to their donor host (‘mismatched’ parasites). All mice were sampled daily, from days 2–6 post infection (PI) at 10:00 UTC to quantify asexual and sexual (gametocytes) stage densities.

### Experiment 2: does the IDC become synchronous in long days?

Experiment 2 tested Arnold’s [34] observation that the IDC of *P. berghei* becomes synchronous in long days. If photoperiod generates a highly synchrony IDC, at any point in the day, infections in long-day hosts (18 h light: 6 h dark) will be more synchronous (i.e. one stage dominating the stage composition) compared to infections in hosts experiencing a standard photoperiod (12 h light: 12 h dark), following [34]. Even synchronization for only part of the IDC should result in a different IDC stage distribution to parasites infecting hosts in the standard photoperiod. WT mice (n = 5 per treatment) were housed in an 18:6 schedule (lights on 16:00; lights off 10:00 UTC). On day 0 PI, infections were initiated from donor mice experiencing the same long-day photoperiod as recipient hosts. On day 6 PI (after 6–7 cycles) mice were sampled at 09:00 UTC and parasites were allocated into 5 morphologically distinct IDC stages (as per Prior et al*.* [11]). These data were compared to previous data from the same Zeitgeber time (ZT17) and the same UTC (09:00), but from hosts housed in a standard 12:12 photoperiod (lights on 07:00; lights off 19:00). These historical infections involved the same mouse and parasite strains, starting dose and sampling days PI, as the long-day infections. Note, the IDC of *P. chabaudi* becomes fully rescheduled to align with host rhythms after perturbations to the phase relationship between host and parasite within 7 cycles so, on top of the 5 cycles in the donor host, this design gave the IDC of *P. berghei* ample opportunity to adjust to a long-day schedule.

### Experiment 3: can host feeding-associated rhythms influence the IDC schedule?

This experiment tested whether the IDC schedule of *P. berghei* can be perturbed by altering the time-of-day that hosts feed [9–11]. TTFL-clock disrupted mice housed in constant darkness were used as hosts to ensure that parasites only experienced rhythms stemming from host feeding-related rhythms. If host feeding-related rhythms influence *P. berghei* in the same manner as *P. chabaudi,* the IDC of *P. berghei* will become synchronous and schizogony will occur during the second half of the window in which hosts feed. *Per1/2*-null (n = 4 per treatment) mice were housed in DD (continuous darkness) with constant dim red LED light. Hosts had access to food constantly (all-day fed) or only during a window of 8 h per day (food in 09:00/food out 17:00 UTC; time restricted fed; TRF). Note, TRF protocols do not lead to caloric restriction or a loss in body mass [10]. Food was provided/removed by changing the cage lid and sweeping the cage for stray pellets at the times of removal and mice in the all-day fed group experienced the same disturbance. On days 5–6 PI, parasites were sampled from 12:00 UTC every 4 h for 28 h. Both the number of parasites at ring-trophozoite stage (“rings”) and the total observed were recorded.

### Experiment 4: do transmission traits show time-of-day variation?

This experiment probed for rhythmicity in reproductive traits that underpin transmission, specifically gametocyte density, exflagellation rate and ookinete density. WT mice (n = 5) experienced a standard photoperiod (lights on 07:00; lights off 19:00 UTC). On day-2 PI, mice were treated with a 30 mg/kg dose of phenylhydrazine hydrochloride (PHZ) via IP injection to induce reticulocytosis and promote gametocyte conversion [38]. On days 5 and 6 post infection, parasites were sampled from each infection at 08:00 UTC (ZT1) and then every 4 h for 24 h. At each sampling point, 2 µl blood samples were diluted in 100 µl ookinete culture media (RPMI-1640 medium containing 10% fetal calf serum, pH 8). After 10 min, a 0.3 µl sub-sample was observed on a haemocytometer and the number of exflagellation events counted over 10 min. At each sampling point, gametocytes were sexed (determined by colour and morphology) and their densities quantified via thin blood smear. Finally, at each sampling point, a second 2 µl blood sample was diluted in 200 µl ookinete culture media, incubated for 24 h at 19 °C and the number of ookinetes in a 0.3 µl sub-sample was counted using a haemocytometer. Exflagellation events and ookinete counts were normalized between samples (exflagellations per male and ookinetes per female) by dividing the counts by the number of male/female gametocytes in the 0.3 µl culture sample they were derived from (gametocytaemia × (RBC density per ml × sample volume)).

### Experiment 5: are oocyst densities influenced by the time-of-day of transmission?

*Plasmodium chabaudi* demonstrates time-of-day variation in infectivity, likely as a consequence of the IDC schedule dictating the age range of gametocytes at the time of transmission [17]. This experiment tested whether *P. berghei* also displays time-of-day variation in infectivity to mosquitoes. If rhythmicity in transmission traits exists and is adaptive (i.e. benefits fitness), parasites transmitted at night are predicted to be more successful. Mosquito cages (6 cages per treatment), each housing 85 female mosquitoes, were randomly allocated to receive blood meals from infected mice (n = 6 per treatment) experiencing their morning (10:00 UTC; ZT3) or evening (20:00 UTC; ZT13) on day 6 PI. This created two groups of infections that varied by time-of-day, for all parties. On day 14 post blood meal, 15 mosquitoes per cage were assessed for oocyst prevalence. Specifically, midguts were dissected, stained for 2 min in 0.5% mercurochrome, washed in PBS and the number of oocysts per midgut counted via microscopy. Circulating gametocyte densities were determined by thin blood smear just prior to mosquitoes feeding on each host.

## Data analysis

The effects of time-of-day, light:dark photoperiod, and host feeding regime on parasite densities and IDC stage proportions were compared between groups using linear mixed-effect models with mouse identity fitted as a random effect. Parasite densities in experiment 1 and gametocyte densities in experiment 5 were analysed using linear models. Oocyst densities in experiment 5 were square root transformed to meet assumptions of normality and homogeneity of variance. Models were selected using step-wise selection via the drop1 function in R. Whether the dynamics of transmission stage metrics in experiment 4 are consistent with ~ 24-h rhythms was assessed using a harmonic regression approach via Circwave (v. 1.4, courtesy of R. Hut; http://www.euclock.org) and confirmed using an alternative non-parametric algorithm via JTK_CYCLE [39]. All other statistical analyses were carried out using R version 3.5.0 (R Foundation for Statistical Computing, Vienna, Austria).

## Results

### Experiment 1: do host rhythms have fitness consequences for P. berghei?

**Fig. 2:**
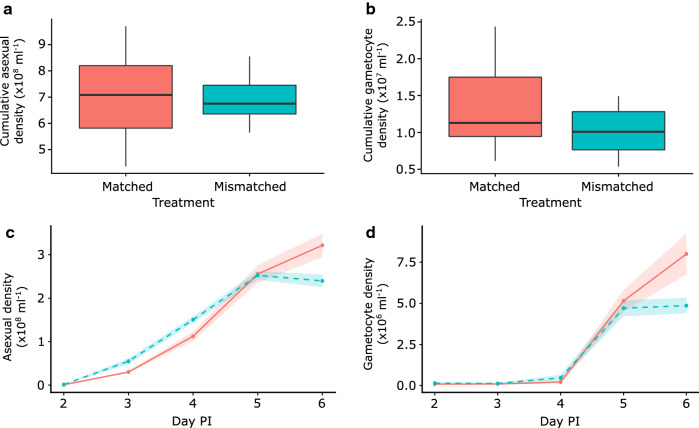
Cumulative (top) and daily (bottom) dynamics for the densities of asexual (**a**, **c**) and sexual stages per ml blood (**b**, **d**). Infections (n = 15) were initiated in wild type MF1 mice with parasites from donor hosts whose rhythms were either ‘matched’ (morning donor/morning recipient) or ‘mismatched’ (morning donor/evening recipient”) to the rhythms of recipient hosts. Shown are (a,b) median parasite densities (black line) and 25–75 percentiles, with whiskers 1.5 times the interquartile range and dots representing outliers and (**c**, **d**) mean with SEM in shading

Overall performance, as measured by cumulative asexual density, did not differ significantly between parasites stemming from donor hosts ‘matched’ or ‘mismatched’ to the timing of rhythms in recipient hosts (Fig. 2a; F_(1,28)_ = 0.10, P = 0.75; mean cumulative asexual density per ml blood × 10^8^ ± SEM = 7.04 ± 0.25). Similarly, cumulative gametocyte density/ml did not differ significantly between ‘matched’ and ‘mismatched’ infections (Fig. 2b; F_(1,28)_ = 3.70, P = 0.06; mean cumulative gametocyte density per ml blood × 10^7^ ± SEM = 1.17 ± 0.09). In the infection dynamics, there was a significant interaction between treatment and time, in which asexual stages (Fig. 2c; treatment:day: $\chi_{4}^{2}$ = 28.57, P < 0.001) and gametocytes (treatment:day: $\chi_{4}^{2}$ = 17.38, P = 0.002; Fig. 2d) varied over time. This is driven solely by the divergence of treatment groups on day 6, in which asexual densities and gametocytes are on average 25% and 40%, respectively, lower in host-mismatched infections (models without day 6 PI; asexual treatment:day: $\chi_{3}^{2}$ = 7.72, P = 0.052, gametocyte treatment:day: $\chi_{3}^{2}$ = 1.55, P = 0.67).

### Experiment 2: does the IDC become synchronous in long days?

**Fig. 3:**
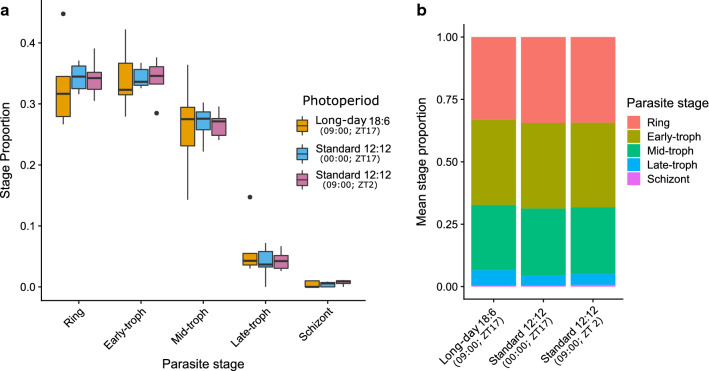
IDC stage distributions for parasites in hosts subjected to a long-day 18:6 light:dark photoperiod vs a standard 12:12 photoperiod. Infections were compared to the stage distributions of infections subjected to a standard 12 h light: 12 h dark photoperiod when sampled at midnight (which is the same ZT as the long-day infections were sampled), and at 09:00 UTC (which is the same UTC as the long-day infections were sampled). **a** Median parasite stage proportions (black line) and 25–75 percentiles, with whiskers 1.5 times the interquartile range and dots representing outliers, and **b** parasite stage distribution illustrated as mean stage proportions. Infections (n = 6) were sampled on days 5–6 PI from wild type MF1 mice

The IDC of parasites in hosts housed in a long-day 18:6 light:dark photoperiod did not become synchronized. Specifically, IDC stage proportions in long-day infections followed the distribution observed previously in standard 12:12 photoperiod infections, which does not differ significantly between morning (ZT2) and evening (ZT17) (Fig. 3, interaction between photoperiod and parasite stage: $\chi_{8}^{2}$ = 5.22, P = 0.73). Parasite stage composition was primarily made up of rings, early trophozoites (trophs) and mid-trophs (mean stage proportion ± SEM: rings = 0.34 ± 0.02, early-trophs = 0.34 ± 0.02, mid-trophs = 0.27 ± 0.02) with late-trophs and schizonts likely sequestering (late-trophs = 0.05 ± 0.01, schizonts = 0.01 ± 0.002).

### Experiment 3: can host feeding-associated rhythms influence the IDC schedule?

**Fig. 4:**
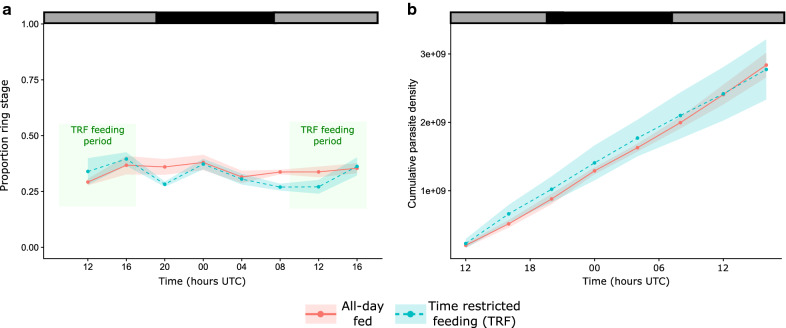
IDC rhythms and cumulative parasite densities in hosts with (TRF) and without (all-day fed) feeding and food-associated rhythms. All hosts were arrhythmic Per1/2-null mice (n = 4), either given access to food continuously (all-day fed; solid line) or during an 8 h window each day (time restricted feeding (TRF); dashed line). Shown are (**a**) the mean ring stage proportion ± SEM in shading and (**b**) mean cumulative parasite density ± SEM in shading (0-28 h sampling period; days 5-6PI). Green boxes in **a** represent period when food is available to TRF mice. All mice were housed in continuous darkness so black and grey bars represent day and night UTC

The IDC did not become synchronized or display altered timing in hosts with strong feeding rhythms (TRF) compared to all-day fed hosts (Fig. 4a). Specifically, the proportion of parasites at ring stage was not significantly affected by time-of-day ($\chi_{1}^{2}$ = 0.31, P = 0.58), host-feeding schedule ($\chi_{1}^{2}$ = 0.92, P = 0.34), or their interaction ($\chi_{1}^{2}$ = 1.37, P = 0.24). The proportion of ring stages remained fairly constant through the 28-h sampling window at 33.5% (± 0.01 SEM). The IDC schedule can also be assessed via the density of developmental stages [40]. Ring stage densities did not differ significantly between all-day fed and TRF mice (host feeding schedule:time interaction: $\chi_{1}^{2}$ = 2.91, P = 0.09 and main effect $\chi_{1}^{2}$ = 1.27, P = 0.26), but ring stage densities did increase over time ($\chi_{1}^{2}$ = 4.66, P = 0.03). This is simply due to replication causing parasite density to increase (specifically, by 80.1% (± 26.8 SEM)) as infections aged during the sampling time series. Thus, cumulative densities varied over the 28 h sampling window (time: $\chi_{1}^{2}$ = 178.08, P < 0.001, treatment: $\chi_{1}^{2}$ = 0.05, P = 0.83), but not in a manner that differed significantly between the TRF and all-day fed hosts (Fig. 4b, interaction $\chi_{1}^{2}$ = 1.26, P = 0.26).

### Experiment 4: do transmission traits show time-of-day variation?

**Fig. 5:**
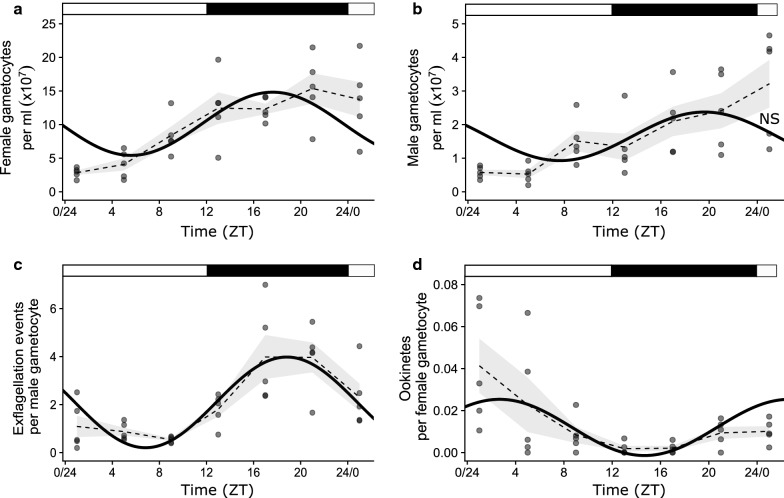
Time-of-day variation in *P. berghei* transmission traits*.* **a** Female gametocyte densities, **b** male gametocyte densities, **c** exflagellation events per male gametocyte and **d** number of ookinetes per female gametocyte. Shown are fitted 24 h Circwave rhythms (solid lines) with mean ± SEM (dashed line and shading) and individual infection data (points). Fits that are non-significant (P > 0.05) are labelled NS and provided for illustration of the trend. Black and white bars represent lights ON (white) and lights OFF (black) and time is given in Zeitgeber time (ZT) in which ZT0 = lights on. Infections (n = 5) were sampled every 4 h on days 5-6PI from wild type MF1 mice pre-treated with phenylhydrazine

Both the densities of male ($\chi_{1}^{2}$ = 34.53, P < 0.001) and female ($\chi_{1}^{2}$ = 36.63, P < 0.001) gametocytes varied during the 24 h sampling window (Fig. 5a, b). For female gametocytes, the pattern is consistent with a 24 h rhythm (Circwave: F_(2,32)_ = 7.01, P = 0.003, JTK_Cycle: BH.Q = 0.04, P = 0.03), with peak density occurring in the evening (ZT17) and a peak-to-trough amplitude of 9.4 × 10^7^ gametocytes per ml. The pattern for males is visually similar but does not fit a 24 h rhythm (Circwave: F_(2,32)_ = 2.77, P = 0.08, JTK_Cycle: BH.Q = 0.20, P = 0.20). In contrast, the number of exflagellation events per male varied during the sampling window (Fig. 5c; $\chi_{1}^{2}$ = 13.57, P < 0.001) and fitted a 24 h rhythm (Circwave: F_(2,32)_ = 19.30, P < 0.001, JTK_Cycle: BH.Q < 0.001, P < 0.001), with ~ 4 × more exflagellation (peak-to-trough amplitude = 3.78 exflagellation events) in the evening (ZT20) than during the day. Similarly, the number of ookinetes per female varied during the sampling window (Fig. 5d; $\chi_{1}^{2}$ = 8.56, P = 0.003), fitting a 24 h rhythm (Circwave: F_(2,32)_ = 5.35, P = 0.01,, JTK_Cycle: BH.Q = 0.003, P = 0.002). Peak density of ookinetes occurred in the morning (ZT 4) rather than the late evening and the rhythm exhibited a peak-to-trough amplitude of 0.03 ookinetes per female.

### Experiment 5: are oocyst densities influenced by the time-of-day of transmission?

**Fig. 6:**
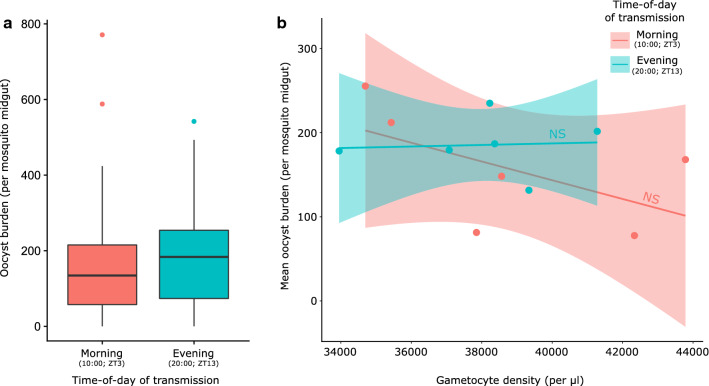
Oocyst burden and gametocyte infectiousness do not vary between morning and evening. Mosquito blood meals occurred when both infected mice and mosquitoes were experiencing their morning (10:00 UTC; ZT3) or their evening (20:00 UTC; ZT13). Shown are (**a**) median oocyst burdens (black line; n = 90) and 25–75 percentiles, with whiskers 1.5 times the interquartile range and dots representing outliers or (**b**) mean oocyst burdens (points; individuals grouped by time-of-day of transmission) with lines indicating non-significant (NS) linear regressions for illustration of trends (SEM in shading; n = 6)

First, there were no significant differences in the densities gametocytes on day 6 PI between infections that were transmitted in the morning (10:00 UTC; ZT3) or the evening (20:00 UTC; ZT13; F_(1,10)_ = 0.17, P = 0.69). Note, hosts in this experiment did not receive PHZ and so, gametocyte densities were lower than in experiment 4. Second, oocyst burden did not differ significantly between morning and evening transmissions, with an overall mean oocyst burden of 171.3 (± 9.84 SEM) oocysts per midgut (Fig. 6a, $\chi_{1}^{2}$ = 0.59, P = 0.44). Furthermore, variation in oocyst burden could not be attributed to variation in gametocyte density (Fig. 6b, $\chi_{1}^{2}$ = 2.95, P = 0.09) or its interaction with the time-of-day of transmission ($\chi_{1}^{2}$ = 0.77, P = 0.38), suggesting that the infectiousness of gametocytes does not vary across the day.

## Discussion

The experiments presented here probe, in several ways, whether the IDC of the asynchronous parasite species *P. berghei,* can be synchronized by perturbations to host rhythms, and whether there are fitness consequences of asynchronous replication. The results reveal that the IDC of *P. berghei* is resistant to being synchronized or scheduled by either long photoperiod days (Fig. 3) or by host feeding-related rhythms (Fig. 4). Furthermore, there is little evidence that host time-of-day affects the within-host component of *P. berghei* fitness. Specifically, the performance of infections (i.e. cumulative densities of asexual stages and gametocytes) is not significantly affected by a “phase-shift” (mismatch) from donor to recipient hosts (Fig. 2). Whilst densities of both asexuals and gametocytes are lower in mismatched infections on day 6 this is unlikely to represent a substantial fitness effect related to host rhythms. First, this drop is not sufficient to affect the cumulative counts. Second, *P. chabaudi* displays a much greater cost of mismatch which is caused by events in the first two cycles that become exacerbated as infections pass through successive cycles of replication [13, 14]. Thus, near identical trajectories for asexual and gametocyte densities until day 6 PI for *P. berghei* is not consistent with a prolonged impact of host rhythms*.* It remains possible that host rhythms impact *P. berghei* and *P. chabaudi* differently, but this requires the host rhythm in question either to be absent from *P. chabaudi* infections or occur 4–5 days sooner than in *P. berghei* infections*.*

The consequences of time-of-day for the between-host (i.e. transmission) component of *P. berghei* fitness is more complicated. Whilst the densities of female gametocytes and the ability of males to exflagellate vary throughout the day with similar patterns, temporal variation in ookinete production follows a damper rhythm with a different pattern (Fig. 5). This suggests that any rhythmicity in the activities of sexual stages is eroded by the time parasites have developed to ookinetes—the ookinete rhythm observed opposes that for males and females and is small, with ookinete prevalence varying only by ~ 2–3% throughout the day. It is unclear what drives the time-of-day variation in the densities of females and exflagellation rates of males. If gametocytes are produced from merozoites stemming from schizogony events occurring at all times-of-day, and gametocytes follow the same developmental rates, then there should be little periodicity in their number or abilities. However, host immune rhythms may influence gametocyte mortality/fertility, imposing rhythms on intrinsically arrhythmic gametocytes. Or perhaps *P. berghei* gametocytes are able to modulate their developmental rate to synchronize maturity. The effects of time-of-day on the densities of females and exflagellation rates of males are small, so may not be biologically relevant, especially by the oocyst stage of transmission, because the time-of-day of transmission does not influence gametocyte infectivity or oocyst density (Fig. 6).

That *P. berghei* fitness is not affected by a “phase-shift” (mismatch) between donor and recipient hosts provides a clue to the costs of mismatch for *P. chabaudi* infections initiated with ring stages*.* For example, *P. chabaudi* infections initiated with mismatched ring stages could perform poorly because hosts mount better defences against evening invaders. If this were the case, the phenomenon should apply to *P. berghei* too, but it does not. This suggests that mis-timing of the IDC itself (e.g. being out of synch with resources needed for development) is costly to *P. chabaudi* from the outset of infection [18]*.* However, the results do not shed light on why the IDC of *P. berghei* is resistant to host time-of-day*.* There are many possible explanations for why a life history trait differs across species. In this case, the explanation depends on whether the asynchronous IDC of *P. berghei* is an adaption (i.e. enhances fitness), is selectively neutral (little effect on fitness), or is a constraint (deleterious but unavoidable). To explore the evolutionary context, it is helpful to consider the IDC as a series of three traits—its level of synchrony, timing of transitions between IDC stages, and the duration of the IDC.

First, how might an asynchronous IDC be an adaption? Faster replication (which enhances competitive ability and within-host survival) is possible from an IDC with a short duration, compared to having an IDC constrained to 24 h (or multiples of) by the need to coordinate with host rhythms. A short IDC is by definition unable to coordinate with 24 h environmental periodicity. Perhaps the benefits of fast replication outweigh the costs of not coordinating with host rhythms, or somehow species with a short IDC are not affected by host rhythms. Perhaps *P. berghei* is able to acquire and store resources through the IDC and so, is not reliant on certain nutrients appearing in the blood when it gets to a certain IDC stage(s)? If so, the question becomes why is *P. chabaudi* unable to achieve this too? An answer might lie in the different within host densities these species reach. Many *P. chabaudi* strains can reach 30–80% peak parasitaemia as late as day 10 PI (depending on starting dose) without host mortality, whereas *P. berghei* tends to kill the host on days 6–8 PI (irrespective of starting dose) due to cerebral malaria, having only reached parasitaemias far lower than 30%. Thus, *P. chabaudi* may require a lot of resources from the host to reach this high biomass, creating a need to efficiently exploit host rhythms, but *P. berghei*’s resource needs might be low enough to be met at any time-of-day. How likely this scenario is, depends on the extent to which development is limited by the resources available within individual RBC versus the blood environment as a whole. For instance, the much greater production of merozoites per schizont by *P. berghei *(6–8 for *P. chabaudi* and 12–18 for *P. berghei*) would intuitively suggest *P. berghei* has greater resource needs from each RBC.

Second, extreme synchrony and extreme asynchrony might be equally good (“alternative”) strategies in a rhythmic environment, with intermediate levels of synchrony being selected against [41]. Synchrony may bring benefits of coordination with host feeding rhythms but be costly in terms of coinciding with rhythmic immune responses that have IDC-stage-specific effects. For example, in human malaria infections, γδ T cells exhibit daily rhythms [42, 43] and effectively target *P. falciparum* merozoites [44]. Asynchrony might protect parasites against immune rhythms but come at the cost of loss of coordination with host feeding rhythms. An asynchronous IDC could also be selectively neutral if *P. berghei* has different resource requirements to *P. chabaudi*, in that the nutrients *P. berghei* needs are not limiting at any time-of-day. Recent work suggests the IDC schedule of *P. chabaudi* is specifically tied to rhythms in blood isoleucine concentration resulting from the host digesting its food [12]. However, amino acid usage patterns in *P. berghei* and *P. chabaudi* are very similar [45], suggesting that if an isoleucine rhythm favours a synchronous and timed IDC in *P. chabaudi*, this should also be the case for *P. berghei*. Perhaps residing in reticulocytes dampens rhythmicity in the resources *P. berghei* needs? Whilst any differences in the ecology of rhythms between *P. berghei* and *P. chabaudi* infections remain unknown, if there are no benefits from a synchronous and timed IDC, natural variation in IDC duration between individual parasites will quickly erode an IDC schedule, perhaps explaining why synchrony is rapidly lost in *P. berghei* infections initiated with a single IDC stage.

Third, *P. berghei* might be under some constraints in murine hosts where it is unable to control its IDC schedule to its detriment. For example, the amplitude of daily rhythms in isoleucine in well fed lab mice may not be sufficient to allow *P. berghei* to tell the time (if *P. chabaudi* is more sensitive to this time cue). This scenario could be tested in 2 ways. First, by probing if the withdrawal of isoleucine from culture media stalls IDC completion of *P. berghei* as it does for *P. chabaudi* and *P. falciparum* [12, 46]. Second, by comparing the performance of asynchronous and artificially synchronized *P. berghei* infections. This is more challenging than it intuitively seems because fitness needs to be assessed within the first few cycles from low density infections before synchrony degrades, and also, the confounding handling effects involved in preparing each type of infection are hard to control for. If asynchronous and synchronous infections can be fairly compared, synchronized infections will perform better if the IDC of *P. berghei* is constrained to be asynchronous.

## Conclusion

The experiments presented here were designed to assess whether host/vector rhythms matter to the IDC of *P. berghei*, rather than explain the ecology underpinning an asynchronous IDC, for which more work is required. This study demonstrates that the IDC of *P. berghei* is resistant to being synchronized and scheduled by environmental photoperiod and by host feeding-related rhythms, and that time-of day has very minor, if any, effects on its fitness. This finding supports recent studies suggesting that across *Plasmodium spp.* features of the IDC schedule are under the control of parasite genes [25–27], rather than directly generated by the host, by for example selectively removing certain IDC stages at certain times of day. Why some species are impervious to the daily rhythms of their hosts and vectors remains mysterious. Further work might benefit from confirming the IDC schedule of *P. berghei* is also asynchronous in the natural rodent host *Grammomys surdaster* (infection dynamics in these rats do those of mirror lab mice [47]) or even bats as *P. berghei* may have a stronger coevolutionary relationship with bats than rodents [48]. Another approach could involve testing whether, unlike *P. chabaudi, P. berghei* has adapted to store resources that are rhythmically provided by the host, thus facilitating IDC completion at any time-of-day. Understanding the costs and benefits of different IDC schedules is central to the success of any interventions that intentionally, or unintentionally, disrupt the timing, synchrony, and duration of the IDC.

## Data Availability

The datasets supporting the conclusions of this article are available in the Edinburgh DataShare repository: https://datashare.ed.ac.uk/handle/10283/3204
